# Case of Spontaneous Popliteal Aneurism

**Published:** 1870-04

**Authors:** 


### Foreign Items.

M. Fleury reports to the *Gazette des Hopitaux* a case of spontaneous popliteal aneurism, treated under his direction, at l'Hotel Dieu de Clermont-Ferrand, by flexion, resulting successfully, after eight days' treatment.

The subject, a cavalry soldier, jarred his right knee in the act of mounting his horse, the left foot being already in the stirrup. The consequences were pain and swelling in the knee and the development of an aneurism as large as an egg. Six months afterward, upon his admission to the hospital, the compressor of Dupuytren was applied, but was discontinued in consequence of the œdema of the limb resulting from its use. The patient, a very intelligent man, in obedience to instructions, kept his leg flexed upon the thigh during several consecutive hours at a time, even when sitting, suspending it only to walk about occasionally. This treatment, continued during eight days, resulted in a complete cure.

M. Verneuil reports to the Imperial Surgical Society, the extraction of three uric-acid calculi, weighing respectively 98 and 27 grammes (weight of smallest not given), by the median operation, from the prostate gland of a patient aged 64 years, followed by complete recovery.— *Gazette des Hopitaux.*

